# Antibody production in mice requires neither vitamin D, nor the vitamin D receptor

**DOI:** 10.3389/fimmu.2022.960405

**Published:** 2022-10-20

**Authors:** Lori A. Plum, William Blaser, Logan Peter, Jean Prahl, Jeremy Seeman, Hector F. DeLuca

**Affiliations:** ^1^ Department of Biochemistry, University of Wisconsin-Madison, Madison, WI, United States; ^2^ Organic Lab, DiaSorin Inc., Stillwater, MN, United States

**Keywords:** vitamin D, antibody, transgenic mice, immunoglobulin, immunity

## Abstract

The vitamin D receptor as well as its ligand have been localized to various immune tissues and cells. These observations have led researchers to hypothesize a role for vitamin D in the immune system. However, a specific role for vitamin D in immunity has yet to be clearly delineated. The work in this report was undertaken to determine if mounting an antibody response is altered in the face of vitamin D-deficiency or when the signaling pathway is eliminated by removal of the nuclear receptor. This investigation provides direct evidence vitamin D is not necessary for producing antibodies, a process paramount for optimal attack against many foreign organisms. The idea that vitamin D plays a significant role in immunity has been proposed repeatedly for many years. To address this important idea we have carried out studies in mice to determine if vitamin D plays a significant role in antibody production. Two animal models were utilized: mice depleted of vitamin D and mice devoid of the vitamin D receptor. Further, a possible role of hypocalcemia resulting from vitamin D deficiency in antibody production was determined. Neither the absence of vitamin D or the vitamin D receptor nor hypocalcemia affected the ability of mice to mount an antibody response to an antigen challenge. Thus, we found no evidence that vitamin D or normal serum calcium is required for this major form of immunity.

## Introduction

It is now well-accepted that 1,25(OH)_2_D_3_, the hormonal form of vitamin D is required for calcium homeostasis (1, 2). The vitamin D hormone acts through a specific nuclear receptor known as the vitamin D receptor (VDR). The liganded receptor binds specific DNA elements to increase or decrease the expression of genes that result in elevation of serum calcium and phosphorus (3). Although it is widely believed that vitamin D plays a role in many organ systems, evidence for this is largely lacking (4). Of these, the immune system has received a great deal of attention. This began with the finding that 1,25(OH)_2_D_3_ binding activity is present in T-lymphocytes (5). Coupled with the epidemiological observation that increased incidence of many immune-based diseases correlate with the distance from the equator, investigation into a possible role of vitamin D in immunity took place (6).

Antibody production is of central importance in immunity; and thus, antibodies are built to detect a large number of antigens. Several classes or isotypes of antibodies are known i.e., IgA, IgD, IgE, IgG and IgM (7). IgA is the most abundant isotype produced in mammals but is primarily present in mucosal secretions (8). IgM is usually the first responder and is produced in the greatest amount upon primary infection (9). A second attack results in large production of IgG and IgA. The other two classes, IgD and IgE, are a very small fraction of the antibodies in circulation. IgE is proposed to be specific to allergic reactions and IgD is believed to be important to the primary response similar to IgM (10, 11). Some of the antibody classes (IgG and IgA) can also have several subtypes expanding the mammalian repertoire of antigen recognition possibilities. Using Keyhole Limpet Hemocyanin (KLH) as the antigen, we have undertaken experiments to determine if vitamin D-deficiency and/or calcium-insufficiency impairs an animal’s ability to mount an antibody response. Our results clearly show that vitamin D and calcium status have no effect on the antibody response to a protein antigen.

## Methods

### Animals

All mice were housed and maintained according to the husbandry procedures adopted by the University of Wisconsin-Madison Biochemistry vivarium and include a 12 hour light-dark cycle, temperature range of 60-72°CF and humidity maintained between 25 and 75%. The mice were group-housed in plastic shoe box cages with stainless-steel wire lids and filter tops. All food and water were provided *ad libitum*. Studies described below were reviewed and approved by the University of Wisconsin-Madison College and Agriculture and Life Sciences Institutional Animal Care and Use Committee.

#### Vitamin D-deficiency study

Male and female C57BL/6J mice (Jackson Laboratories; Stock No. 000664) were born and raised under vitamin D-deficient conditions. The parents of these mice were bred in an animal room in which filter sleeves were placed over the ceiling bulbs to block UV light and they were maintained on a purified diet lacking vitamin D (12). Weanlings were fed a vitamin D-deficient diet containing high calcium (2%), high phosphorus (1.25%) and 20% lactose. At approximately 85 days of age (age range: 56-113), the mice were divided into 3 groups (n=10; 6 females and 4 males) and fed the following diets:

Group 1: D-deficient – 0.47% calcium/0.3% phosphorus for 3 weeks followed by vitamin D deficient 0.235% calcium/0.3% phosphorus for the duration of the study; (-D) Low Ca

Group 2: D-deficient – 0.87% calcium/0.3% phosphorus; (-D) Normal Ca

Group 3: D-deficient moved to the +D 0.87% calcium diet at the start of the study; (-D) to (+) D

A fourth group of age-matched animals [(+D); range 89-111 days] used in this study comprised a mixture of male and female mice born in our facility under D-sufficient conditions. These mice were fed a vitamin D containing diet with high calcium (2%) and phosphorus (1.25%) in addition to 20% lactose. At approximately 106 days of age, they were switched to the same diet but containing lower amounts of calcium and phosphorus (0.87% and 0.3%, respectively) and no lactose for the duration of the study.

Blood was collected at various times to confirm vitamin D status and calcium levels. Vitamin D status was determined by measuring 25(OH)D_3_ and 1,25(OH)D_3_ levels using the antibody-based assay by Diasorin (Liaison 25 OH Vitamin D Total Assay Stillwater, MN). Serum calcium levels were determined by atomic absorption spectrometry (Perkin Elmer Model PinAAcle 500).

When the animals were 129-148 days of age, each received an intraperitoneal injection (100 μL) of Keyhole Limpet Hemocyanin (0.025 mg/ml; Sigma, Cat. No. H7017). The KLH emulsion was prepared by mixing a 5 mg/ml aqueous solution of KLH with PBS and Complete Freund’s Adjuvant (Sigma, Cat. F5881). A booster injection at one third the initial concentration was administered 30 days after the first injection. At multiple times following each antigen injection, blood was collected to determine antibody amount by ELISA assay (see below).

#### VDR knockout study

Male, VDR knockout mice (B6.129S4-*Vdr^tm1Mbd^*/J) and wild-type littermates (n= 4-8/genotype/dietary group) were generated in our vivarium from breeder stock obtained from Jackson Laboratories (Stock No. 006133). These mice were fed a diet high in calcium (2%) and phosphorus (1.25%) with 20% lactose until the study start. Then at approximately 113 days (range 100-135) days of age, they were switched to purified diet containing either 0.87% calcium and 0.3% phosphorus (Normal Calcium) or 0.235% calcium and 0.3% phosphorus (Low Calcium) for the duration of the study. The mice received the KLH injection between 135-170 days of age and a booster injection at one third concentration 37 days after the first one. Blood was collected at various timepoints for antibody analysis using the ELISA procedure described below and calcium determination described above.

### ELISA procedure

Costar 96 well plates (Corning Cat No. 3361) were coated with 0.2 µg KLH (diluted in 0.1 M sodium bicarbonate solution) overnight at 4˚C and incubated 2 hours with blocking buffer (PBS, 0.5% BSA) at room temperature (RT). After blocking, the wells were washed with PBS, 0.05% Tween-20 three times (total volume 1050 µL) and the standards ((purified anti-KLH; Biolegend (IgG1) Cat No. 408502) or BD Pharmingen (IgM) Cat No. 550340 or pooled blood samples (IgA)) and experimental serum samples (range of dilutions from 1:10 to 1:750,000) were added in 100 µL of PBS, 0.25% BSA and 0.025% Tween-20. After 1.5 hours of incubation at RT, the standards and serum samples were removed and the wells washed three times (total volume 1050 µL) with PBS, 0.05% Tween-20. Then 100µL goat anti-mouse IgG, IgG1, IgG3, IgM or IgA conjugated to HRP (Abcam Cat No. ab97265, ab97240, ab97260, ab97230 and ab97235, respectively) was added (1:10000; except IgG3 and IgA were added at 1:220) and incubated for another hour at RT followed by 3 washes (total volume 1050 µL) of PBS, 0.05% Tween-20. After the washes, 100 µL of substrate (1-Step™ Turbo TMB-ELISA; Thermo Scientific, Cat. No. 34022) was added and incubated for 30 minutes at RT. The reaction was stopped with 100µL 2N H_2_SO_4_ and then immediately analyzed at 450 nm using a Biotek Synergy H1 plate reader. All values were calculated based on the standard curve run on every plate. Affinity purified IgG1 was used to generate standard curves for all IgG analyses: total IgG, IgG1 and IgG3. Affinity purified IgM was used to generate the standard curve for the IgM analyses. Since we were unable to secure a commercial preparation of purified IgA, a pooled mouse serum sample was used to generate the standard curves for the IgA analyses. The coefficient of variation (intra-assay) for all assays ranged from 1.9 to 9.7%. Note: No units are provided on the Y-axis of the graphs because the values (amount of antibody/volume of blood) should not be compared across graphs given the manner in which the values were derived. The only comparisons that can be made are across dietary treatment or genetic groups for a given antibody type.

### Statistical analyses

Statistical analyses were developed and performed under the guidance of the University of Wisconsin-Madison CALS Statistical Consulting Group. Analysis of serum calcium was done using ANOVA followed by Tukey’s *post-hoc* tests. The area under the curve for each antibody isotype or subclass response was analyzed for each dietary group and/or genotype. Normal distribution of the data was found. Comparisons were made within the primary and secondary responses and across both responses using the generalized linear model. To address missing data points, multiple imputation using multivariate distribution was used. Fifty datasets were imputed.

## Results

Keyhole Limpet Hemocyanin (KLH) is a large protein that is often used for the purposes of generating monoclonal antibodies because of its potent antigenicity. Due to this property, it was selected as the antigen to study the impact of vitamin D deficiency with or without calcium insufficiency on antibody production in mice. Mice were first depleted of vitamin D as described in the Methods section. Once depleted, they were fed a D-deficient diet that either maintained low serum calcium (-D Low sCa) or normalized blood calcium levels (-D, Norm sCa), see **Table 1**. In addition, one group was restored to normal vitamin D levels and a fourth group was never depleted of vitamin D, see **Table 1**.

**Table 1:** Serum parameters in vitamin D-deficiency study.

	Serum Ca			Serum 25(OH)D_3_		Serum 1,25(OH)_2_D_3_	
	mg/dL			ng/mL		pg/mL	
Dietary Groups	*Initial*	*Day 32*	*Final*	*Initial*	*Final*	*Initial*	*Final*
(-) D Low Ca	8.3 ± 0.1^a^	7.4 ± 0.5^a,b^	6.4 + 0.3^*^	<4	<4	<5	<5
(-) D Normal Ca	8.7 ± 0.1^b^	8.8 ± 0.4	9.1 + 0.2	<4	<4	<5	<5
(-) D to (+) D	9.3 ± 0.2^a,b^	9.3 ± 0.1^a^	9.4 + 0.3	29 ± 4	35 ± 3	<5	<5
(+) D	8.8 ± 0.2	10 ± 0.4^b^	8.8 + 0.2	28 ± 4	31 ± 4	<5	<5

Statistical significance (p < 0.05) is denoted by superscript letters or asterisk. An asterisk indicates the group was significantly different from all other groups. Statistical analyses were not done for serum 25(OH)D_3_ or 1,25(OH)_2_D_3_ measurements.

**Figure 1:**
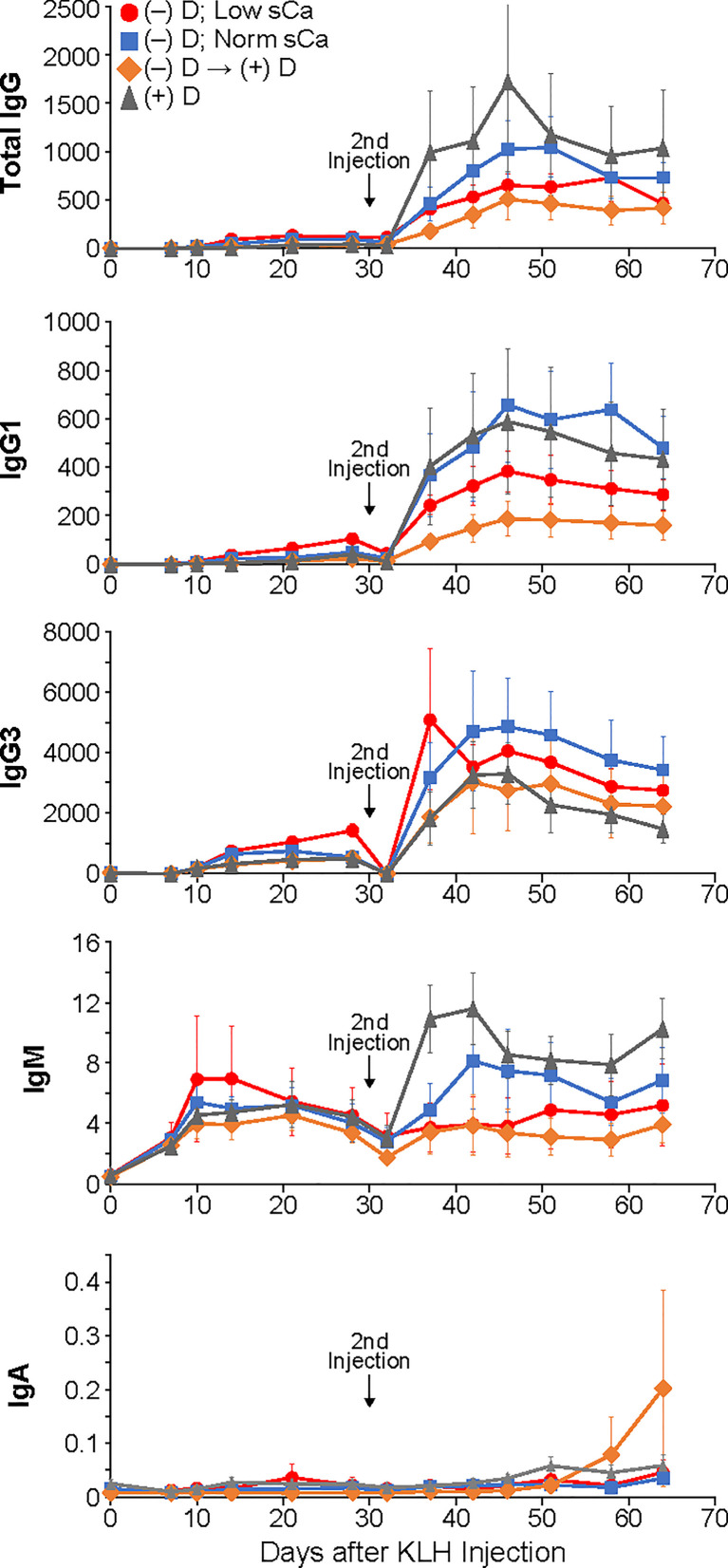
Primary and secondary antibody responses for Isotypes IgG, IgM and IgA and subclasses IgG1 and IgG3 in mice made vitamin D-deficient through dietary means. Statistical analyses were done as described in the Methods with only a few minor differences detected as noted in the text. The error bars are SEM.

After the first injection of KLH, a small but measurable increase in antibody production was observed in all dietary groups (**Figure 1**). The only statistically significant change in total IgG levels during the primary response was in the D-deficient group on low calcium compared to the D-sufficient group where D-deficiency and hypocalcemia combined actually caused a stronger antibody response (**Figure 1A**). Similar to the total IgG results, there was no impact of vitamin D or calcium status on the generation of the lower percentage subclasses unless both vitamin D-deficiency as well as low calcium were experienced, then stronger IgG1 and IgG3 responses were observed (**Figures 1B, C**). The levels of IgM and IgA during the primary response were not significantly different amongst any of the dietary groups (**Figures 1D, E**).

As expected, the second injection of KLH resulted in a very large boost in antibody production for all isotypes and subclasses that was statistically different from the primary response (**Figure 1**). However, no differences were seen in total IgG production or any of the less populous subclasses, IgG1 and IgG3, or isotype IgA. However, in contrast to the primary response, the levels of IgM did show statistically significant change during the secondary response. Animals that were both D-deficient as well as hypocalcemic had lower IgM levels compared to mice on a D-sufficient diet their entire life. Oddly, animals that were made D-deficient and then restored to normal D levels produced the same amount of IgM antibodies as the animals with depleted vitamin D and calcium. Yet the animals that were D-deficient but with restored calcium were no different than D-sufficient animals. No differences between male and females were found.

Because of the variable, graded nature of making animals vitamin D-deficient, a second, genetically altered animal model was utilized. Mice in which the vitamin D receptor was eliminated were compared to wildtype (WT) littermates. In addition, some animals were maintained on a diet that keeps the blood calcium low in the receptor-less mice; while others were given a diet to restore normal blood calcium levels (**Table 2**).

**Table 2:** Serum parameters in VDR knockout study.

	Serum Ca		
		mg/dL	
	*Initial*	*Day 33*	*Final*
VDR KO Low Ca	6.1 ± 0.2*	5.9 ± 0.1*	4.5 ± 0.3*
VDR KO Normal Ca	8.4 ± 0.2	9.9 ± 0.1	8.4 ± 0.1
VDR WT Low Ca	8.2 ± 0.2	9.4 ± 0.2	8.0 ± 0.2
VDR WT Normal Ca	9.2 ± 0.2	10.1 ± 0.3	8.9 ± 0.1

Statistical significance (p < 0.05) is denoted by an asterisk. An asterisk indicates the group was significantly different from all other groups.

**Figure 2:**
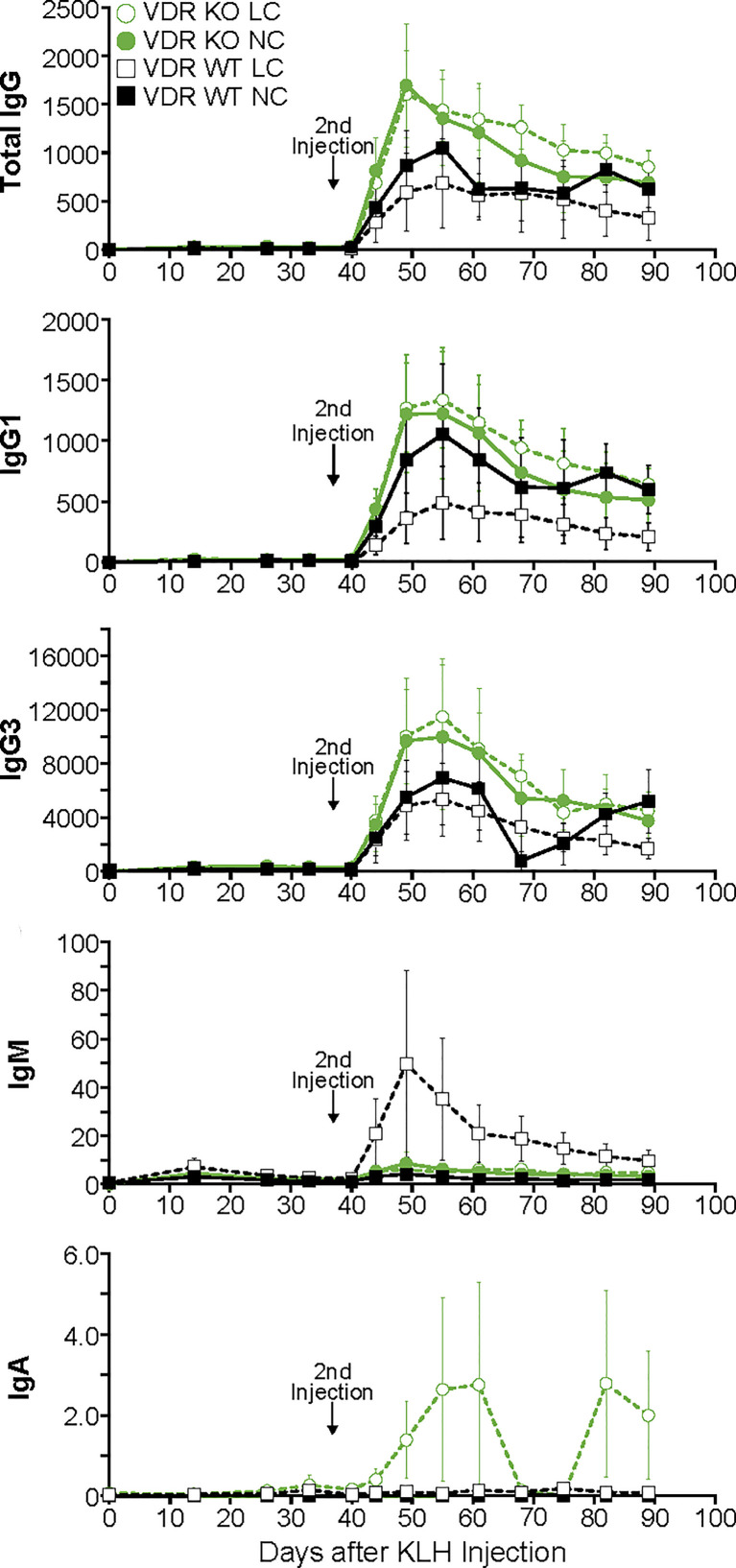
Primary and secondary antibody responses for Isotypes IgG, IgM and IgA and subclasses IgG1 and IgG3 in mice devoid of the vitamin D receptor compared to wild-type littermates. Statistical analyses were done as described in the Methods with only a few minor differences detected as noted in the text. The error bars are SEM.

The pattern of antibody production in response to the KLH antigen in VDR knockout mice was essentially the same as WT mice both during the primary response as well as the secondary response (**Figure 2**). The only statistically significant difference observed was in the production of IgG1 antibodies during the secondary response in animals on a low calcium diet where the VDR knockout animals had a higher level than the WT animals. Furthermore, there was no impact of low blood calcium on the animal’s ability to mount a response. These results held up whether total IgG was analyzed or the individual populations of IgG1 or IgG3. In addition, no effect on the amount of isotype IgM or IgA antibodies was observed.

## Discussion

These results demonstrate that the vitamin D system does not play a significant role in an animal’s ability to mount an antibody response to an antigen challenge in the form of a protein. Whether mice were made deficient in vitamin D, or lacked the vitamin D receptor, the generation of antibodies against KLH remained largely unaffected. Of the few statistically significant changes that were detected, none were present in both animal models.

While there are multiple steps and facets for fighting infection, antibody production is a key component for battling many foreign organisms. Consistent with our findings are reports indicating rodent, normal resting human B cells and many B lymphocyte cell lines lack VDR (13–15). However, it should be noted that others have reported VDR to be present in cultured B cells, whether activated or not, highlighting the need for *in vivo* data to provide the most meaningful information (16–18). Inconsistent with our findings is a report showing a significant reduction in IgG1 antibodies 14 or 28 days after *C. rodentium* infection of mice lacking the enzyme necessary for production of 1,25(OH)_2_D_3_ or in animals made deficient in vitamin D through dietary means (19). This potential inconsistency might be due to the difference of the insult on the immune system as not all infectious organisms are fought using the exact same mechanisms, including in some cases, no requirement for antibodies (20–22). However, as shown in this study, the antibody response amongst animals varies significantly over time, and assessment at only one or two timepoints could provide a different answer than area under the curve comparisons collected over the entire antibody production lifespan, a strength of the current investigation.

IgE levels were not analyzed in the current studies since they were not expected to change with the immunization conditions used here (23). However, a study done in mice lacking VDR or deficient in vitamin D showed double the levels of circulating IgE compared to WT littermates or vitamin D-sufficient control animals, respectively (24). Whether or not these elevations of basal amounts of IgE translate to a significant response when faced with a triggering event remains to be determined.

Certainly fighting infections involves more than just B cells and antibodies, and several laboratories have demonstrated a plausible role for vitamin D in macrophages and T cells using cultured cells (25). In these studies, both macrophages and T cells are shown to harbor the nuclear receptor for vitamin D, as well as the enzyme necessary for local production of 1,25(OH)_2_D_3_. Increased generation of the vitamin D hormone leads to increased production of antimicrobial peptides which directly attack foreign invaders. This proposed function has yet to be confirmed *in vivo*. In addition, there is accumulating *in vivo* evidence in rodents that vitamin D-deficiency is not detrimental to fighting infection, whether viral, parasitic or bacterial in nature (26–28). In fact, one report has shown the opposite, where animals made vitamin D-deficient battle infections better than rodents with normal levels of vitamin D (29).

Due to the well-established function of vitamin D hormone in maintaining normal blood calcium levels, animals made vitamin D- or receptor-deficient, have very low blood calcium concentrations unless placed on a high calcium diet. The studies shown here demonstrate low blood calcium does not significantly affect antibody production. This finding contrasts with the observations that low calcium exacerbates several autoimmune based diseases (30, 31). In addition, there is a large body of work detailing the importance of calcium in dictating the pathogenicity of microorganisms (32). While there is strong evidence calcium plays a significant role in maintaining a healthy immune system, it may act outside of B-cell production of antibodies.

Rodents have been successfully utilized for antimicrobial drug research for many decades resulting in antibiotics invaluable for treating human disease (33). But recently, mouse and rat models have been questioned for use in vitamin D based investigations of infections because genomic analysis of three antimicrobial peptide (AMP) genes appear to lack the same vitamin D regulatory element present in higher organisms (34). However, vitamin D regulation of these AMPs in higher organisms has yet to be shown *in vivo*. Furthermore, there are many AMPs available for attacking infectious agents and it is possible that those regulated by vitamin D differ between rodents and humans (35). One advantage of using rodents is that they can be depleted of vitamin D to maximize the ability of detecting a response. In addition, they can be genetically manipulated to assist in interpretation and understanding of the mechanism(s) of action.

The idea of using vitamin D therapy *via* cod liver oil for infections originated with tuberculosis (TB) patients in the 1800’s (36). Since that time, numerous clinical studies have been completed in patients with TB and other respiratory diseases. A couple of meta-analyses indicate vitamin D intervention positively alters acute respiratory tract infection, particularly in those individuals with the most deficient levels of 25(OH)D (37, 38). However, a very large placebo controlled trial conducted in countries where individuals tend towards low vitamin D status, did not support the meta-analysis findings (39). In addition, multiple studies assessing the relationship of vitamin D status to antibody production after various types of vaccinations, including the mRNA vaccine to SARS-CoV-2, did not find any indication that response was related to 25(OH)D status (40–42). These clinical trials showing no impact of vitamin D are fully supported by the experimental data reported here where production of IgM, IgG and IgA antibodies in the serum are not altered in the face of vitamin D ligand insufficiency or receptor elimination even when accompanied by low calcium levels.

## Data availability statement

The raw data supporting the conclusions of this article will be made available by the authors, without undue reservation.

## Ethics statement

The animal study was reviewed and approved by Research Animal Resources Committee of the College of Agricultural and Life Sciences, University of Wisconsin–Madison.

## Author contributions

LAP – wrote the paper, designed the experiments and reviewed the data; WB – carried out some of the animal experiments and performed the statistical analysis; LP and JP - carried out some of the animal experiments; JS – performed the 25(OH)D_3_ and 1,25(OH)_2_D_3_ analyses; HD – developed the overall idea and edited the paper. All authors contributed to the article and approved the submitted version.

## Funding

This work was supported by a fund from the Wisconsin Alumni Research Foundation.

## Acknowledgments

We thank Mindy Kendrick and students, Colin Schuh, Callie Wessel and Sydney Eftemoff, for their assistance with the nearly 500 ELISA assays.

## Conflict of interest

Author JS was employed by DiaSorin Inc.

The remaining authors declare that the research was conducted in the absence of any commercial or financial relationships that could be construed as a potential conflict of interest.

## Publisher’s note

All claims expressed in this article are solely those of the authors and do not necessarily represent those of their affiliated organizations, or those of the publisher, the editors and the reviewers. Any product that may be evaluated in this article, or claim that may be made by its manufacturer, is not guaranteed or endorsed by the publisher.
